# Supplementary material for: Adaptive generalization in pollination systems: Hawkmoths increase fitness to long‐tubed flowers, but secondary pollinators remain important
Source: Ecol Evol. 2024 May 22;14(5):e11443. doi: 10.1002/ece3.11443 (PMC11112297; doi:10.1002/ece3.11443)
Supplement: Supplementary file 1 — Data S1. [file ECE3-14-e11443-s002.pdf]

**Supplemental Materials**

## SUPPLEMENT

**Table S3**. Pollinator visitation data for focal populations by count of visits, proportion of total visits, and visitation rate (number of visits/ flower/ hour).

**Table S3. Pollinator visitation to exclusion experiment populations**

| | | | | | **Count** | | | | |
|---|---|---|---|---|---|---|---|---|---|
| Population | Year | Dataset | Other diurnal | Hawkmoth | Small/ med bees | Other nocturnal | Bumble-bee | Humming-bird | Total visits |
| SCC | 2012 | Narrow view | 1 | 88 | 30 | 0 | 0 | 0 | 119 |
| SILB12 | 2012 | Narrow view | 0 | 34 | 0 | 3 | 0 | 0 | 37 |
| SILB13 | 2013 | Narrow view | 0 | 0 | 3 | 0 | 0 | 0 | 3 |
| CQL | 2019 | Narrow view | 0 | 0 | 1 | 0 | 0 | 0 | 1 |
| CQL | 2019 | Wide view | 0 | 0 | 7 | 0 | 67 | 29 | 103 |
| LVH | 2019 | Narrow view | 0 | 0 | 1 | 0 | 0 | 3 | 4 |
| LVH | 2019 | Wide view | 5 | 3 | 1 | 2 | 0 | 44 | 55 |
| SBL | 2019 | Narrow view | 0 | 4 | 0 | 0 | 0 | 0 | 4 |
| SBL | 2019 | Wide view | 0 | 29 | 0 | 0 | 0 | 0 | 29 |
| SCL | 2019 | Narrow view | 0 | 46 | 2 | 0 | 0 | 0 | 48 |
| SCL | 2019 | Wide view | 0 | 16 | 0 | 0 | 2 | 0 | 18 |
| SIC | 2019 | Narrow view | 8 | 53 | 20 | 0 | 0 | 0 | 81 |
| SIC | 2019 | Wide view | 3 | 49 | 0 | 0 | 0 | 0 | 52 |
| SMP | 2019 | Narrow view | 0 | 1 | 0 | 0 | 0 | 0 | 1 |
| SMP | 2019 | Wide view | 0 | 0 | 0 | 0 | 105 | 0 | 105 |
| SNRM | 2019 | Narrow view | 1 | 0 | 165 | 0 | 62 | 0 | 228 |
| SNRM | 2019 | Wide view | 0 | 0 | 7 | 0 | 14 | 0 | 21 |

## Supplemental Materials

| | | | Proportion | | | | | |
|---|---|---|---|---|---|---|---|---|
| Population | Year | Dataset | Other diurnal | Hawkmoth | Small/ medium bees | Other nocturnal | Bumble-bee | Humming-bird |
| SCC | 2012 | Narrow view | 0.008403 | 0.739496 | 0.252101 | 0 | 0 | 0 |
| SILB12 | 2012 | Narrow view | 0 | 0.918919 | 0 | 0.081081 | 0 | 0 |
| SILB13 | 2013 | Narrow view | 0 | 0 | 1 | 0 | 0 | 0 |
| CQL | 2019 | Narrow view | 0 | 0 | 1 | 0 | 0 | 0 |
| CQL | 2019 | Wide view | 0 | 0 | 0.067961 | 0 | 0.650485 | 0.2815534 |
| LVH | 2019 | Narrow view | 0 | 0 | 0.25 | 0 | 0 | 0.75 |
| LVH | 2019 | Wide view | 0.090909 | 0.054545 | 0.018182 | 0.036364 | 0 | 0.8 |
| SBL | 2019 | Narrow view | 0 | 1 | 0 | 0 | 0 | 0 |
| SBL | 2019 | Wide view | 0 | 1 | 0 | 0 | 0 | 0 |
| SCL | 2019 | Narrow view | 0 | 0.958333 | 0.041667 | 0 | 0 | 0 |
| SCL | 2019 | Wide view | 0 | 0.888889 | 0 | 0 | 0.111111 | 0 |
| SIC | 2019 | Narrow view | 0.098765 | 0.654321 | 0.246914 | 0 | 0 | 0 |
| SIC | 2019 | Wide view | 0.057692 | 0.942308 | 0 | 0 | 0 | 0 |
| SMP | 2019 | Narrow view | 0 | 1 | 0 | 0 | 0 | 0 |
| SMP | 2019 | Wide view | 0 | 0 | 0 | 0 | 1 | 0 |
| SNRM | 2019 | Narrow view | 0.004386 | 0 | 0.723684 | 0 | 0.27193 | 0 |
| SNRM | 2019 | Wide view | 0 | 0 | 0.333333 | 0 | 0.666667 | 0 |

## Supplemental Materials

| Population | Year | Dataset | Visitation rate (visits/ flower/ hour) Other diurnal | Hawkmoth | Small/ medium bees | Other nocturnal | Bumble-bee | Humming-bird |
|---|---|---|---|---|---|---|---|---|
| SCC | 2012 | Narrow view | 0.001484 | 0.130628 | 0.044532 | 0 | 0 | 0 |
| SILB12 | 2012 | Narrow view | 0 | 0.04629 | 0 | 0.004084 | 0 | 0 |
| SILB13 | 2013 | Narrow view | 0 | 0 | 0.001938 | 0 | 0 | 0 |
| CQL | 2019 | Narrow view | 0 | 0 | 0.000995 | 0 | 0 | 0 |
| CQL | 2019 | Wide view | 0 | 0 | 0.000665 | 0 | 0.006361 | 0.0027531 |
| LVH | 2019 | Narrow view | 0 | 0 | 0.001948 | 0 | 0 | 0.0058442 |
| LVH | 2019 | Wide view | 0.001513 | 0.000908 | 0.000303 | 0.000605 | 0 | 0.0133148 |
| SBL | 2019 | Narrow view | 0 | 0.007084 | 0 | 0 | 0 | 0 |
| SBL | 2019 | Wide view | 0 | 0.060421 | 0 | 0 | 0 | 0 |
| SCL | 2019 | Narrow view | 0 | 0.085928 | 0.003736 | 0 | 0 | 0 |
| SCL | 2019 | Wide view | 0 | 0.0156 | 0 | 0 | 0.00195 | 0 |
| SIC | 2019 | Narrow view | 0.011483 | 0.076077 | 0.028708 | 0 | 0 | 0 |
| SIC | 2019 | Wide view | 0.000979 | 0.015985 | 0 | 0 | 0 | 0 |
| SMP | 2019 | Narrow view | 0 | 0.000408 | 0 | 0 | 0 | 0 |
| SMP | 2019 | Wide view | 0 | 0 | 0 | 0 | 0.005737 | 0 |
| SNRM | 2019 | Narrow view | 0.001082 | 0 | 0.178571 | 0 | 0.0671 | 0 |
| SNRM | 2019 | Wide view | 0 | 0 | 0.007799 | 0 | 0.015597 | 0 |

**Supplemental Materials**

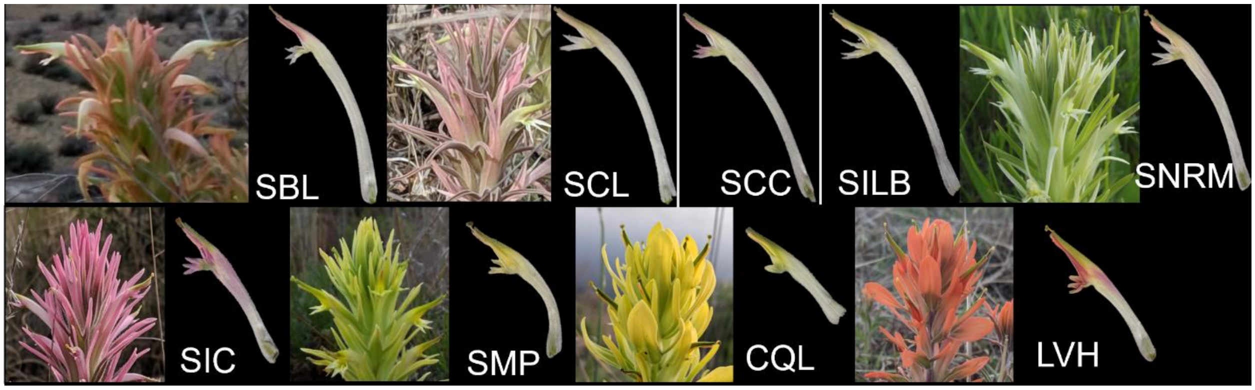


**Figure S1.** Representative photos of floral morphs and corollas for populations where exclusion experiments were conducted. Top row: long-tubed populations; bottom row: short-tubed populations. First letter of population codes denotes species: S = *C. sessiliflora*, C = *C. citrina*, L = *C. lindheimeri*. Images not to scale.

**Supplemental Materials**

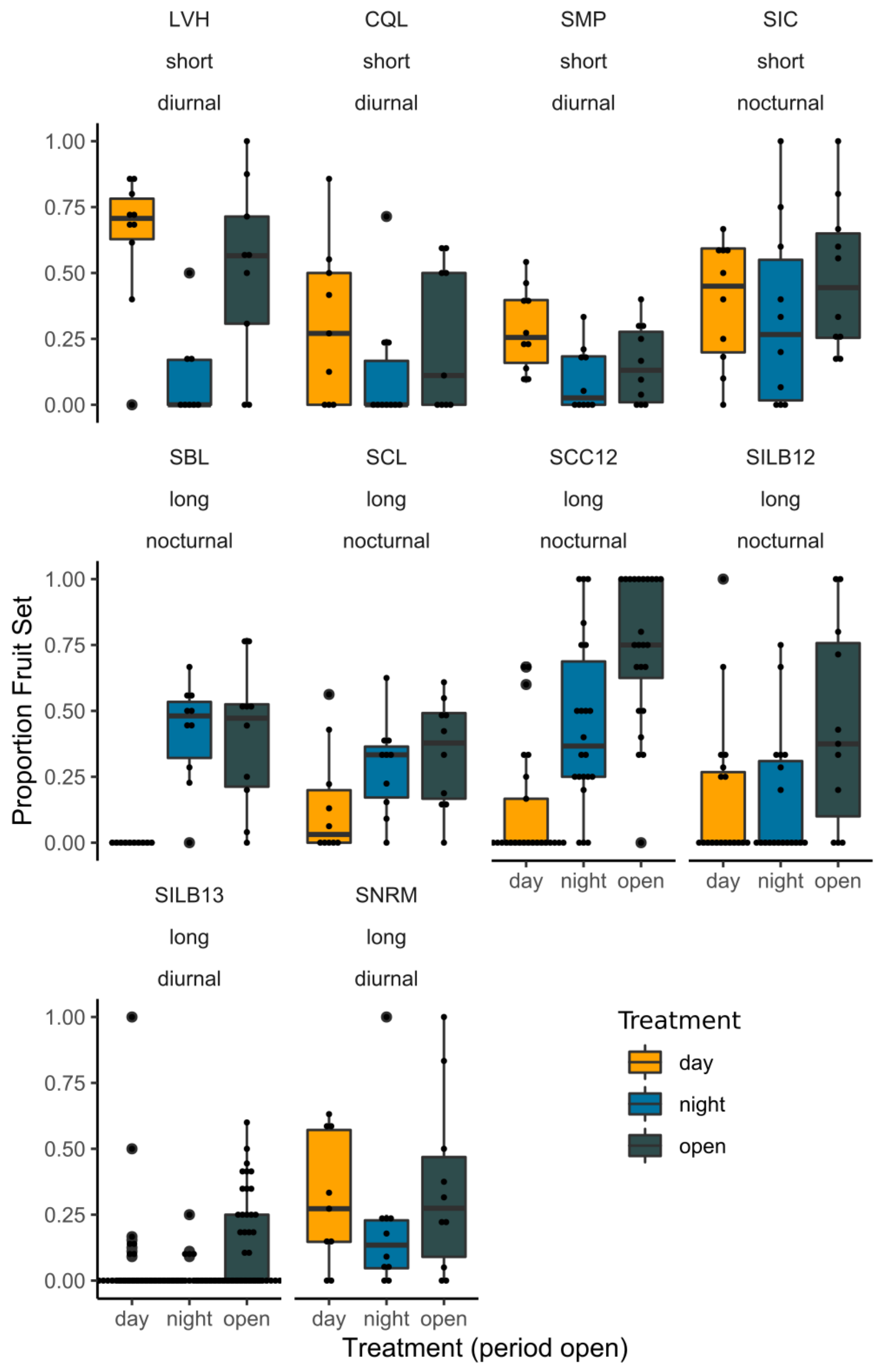


**Figure S2.** Population-level results of the pollinator exclusion experiment performed in nine natural populations. Proportion fruit set is shown for each treatment: plants open to pollinators during the day, at night, or fully open. Population codes are given at the top of each plot. Plots are labelled with the corolla length morph of the population and whether the most frequent visitor to the population during experimental windows was diurnal or nocturnal (i.e., hawkmoths). The exclusion experiment was repeated in subsequent years at SILB (2012 and 2013).
