# Supplementary material for: Adaptive generalization in pollination systems: Hawkmoths increase fitness to long‐tubed flowers, but secondary pollinators remain important
Source: Ecol Evol. 2024 May 22;14(5):e11443. doi: 10.1002/ece3.11443 (PMC11112297; doi:10.1002/ece3.11443)
Supplement: Supplementary file 2 — Appendix S1. [file ECE3-14-e11443-s001.docx]

APPENDIX

**Appendix 1. Breeding system of *Castilleja sessiliflora***

**Table S1.** Fruit set to plants growing in a common garden that were either fully bagged to exclude pollinators and unmanipulated (“bagged” flowers) or bagged and hand-pollinated with self-pollen (“hand-selfed” flowers). Plants were grown at Chicago Botanic Garden from seed wild-collected from populations across the range. Tests were conducted in 2019.

| Population | State | Exclusion experiment population? | No. of plants | No. of flowers bagged | No. of fruits to bagged flowers | Bagged fruit set (percent) | No. of flowers hand self-pollinated | No. of fruits to hand-selfed flowers | Hand-selfed fruit set (percent) |
| --- | --- | --- | --- | --- | --- | --- | --- | --- | --- |
| SMP | TX | Yes | 1 | 29 | 0 | 0.00% | 12 | 0 | 0% |
| SCC | CO | Yes | 1 | 9 | 0 | 0.00% | 3 | 0 | 0% |
| SDC | CO | No | 2 | 19 | 0 | 0.00% | 8 | 0 | 0% |
| SILB | IL | Yes | 1 | 35 | 1 | 2.86% | 6 | 0 | 0% |
| SFP | MN | No | 1 | 13 | 0 | 0.00% | 4 | 0 | 0% |
| **Total** |  |  | **6** | **105** | **1** | **0.95%** | **33** | **0** | **0%** |

**Table S2.** Fruit set to plants growing in natural populations that were fully bagged to exclude pollinators during pollinator exclusion experiments conducted in 2012 and 2013.

| Population | State | Year | No. of plants | No. of flowers bagged | No. of fruits to bagged flowers | Bagged fruit set (percent) |
| --- | --- | --- | --- | --- | --- | --- |
| SCC | CO | 2012 | 24 | 91 | 3 | 3.30% |
| SDC | CO | 2012 | 24 | 69 | 0 | 0.00% |
| SILB | IL | 2012 | 6 | 26 | 0 | 0.00% |
| SILB | IL | 2013 | 28 | 311 | 0 | 0.00% |
| **Total** |  |  | **82** | **497** | **3** | **0.60%** |
